# Gut microbiome in alcohol-associated liver disease: interactions and therapeutic strategies

**DOI:** 10.3389/fphar.2026.1770833

**Published:** 2026-04-01

**Authors:** Xianting Liang, Junning He, Qiuting Wu, Lixiang Fu, Yongfang Liu

**Affiliations:** 1 Department of Infectious Diseases, The Third People’s Hospital of Chengdu, Chengdu, China; 2 Department of Clinical Medicine, North Sichuan Medical College, Nanchong, China; 3 Basic Medical College, Chengdu University of Traditional Chinese Medicine, Chengdu, China

**Keywords:** alcohol-associated liver disease (ALD), bacteriophage therapy, biomarkers, fecal microbiota transplantation (FMT), gut microbiome, gut-liver axis, multi-omics analysis, probiotics

## Abstract

Alcohol-associated liver disease (ALD), a significant cause of chronic liver disease worldwide, is strongly linked to gut microbiome dysregulation. Heavy alcohol use disrupts the gut bacterial equilibrium and damages the intestinal barrier, making it more permeable to microbial toxins (e.g., endotoxins) that trigger liver inflammation. Many studies have investigated ALD, but no single microbial marker has yet been identified as diagnostic. Results from microbiome studies on this condition have been inconsistent; consequently, scientists are developing new microbiome-based indices and multi-omics approaches to improve their ability to predict diseases. The review evaluates current findings on how disturbances in the gut microbiome and deterioration of the intestinal barrier contribute to the development of ALD. The assessment includes microbiome-based treatments such as probiotics, fecal microbiota transplantation (FMT), and bacteriophage therapy. Research indicates that probiotics and FMT treatments may enhance liver function and reduce inflammation in patients with ALD. The studies present conflicting results because researchers used different methods and worked with limited numbers of participants. Bacteriophage therapy exists as an experimental treatment method. The development of personalized microbiome treatments, along with biomarker standardization and solutions to technical and ethical challenges, will enable these strategies to enter medical practice. The review integrates existing knowledge of the gut-liver axis in ALD to demonstrate the clinical potential of microbiome-based treatments while highlighting the need for additional research to enhance treatment outcomes.

## 1 Introduction

The worldwide occurrence of chronic liver diseases results in approximately 2 million deaths annually, accounting for about 4% of all deaths globally (Manikat et al., 2024). The incidence of alcohol-associated liver disease (ALD) has risen, making it a major cause of chronic liver disease (Wang et al., 2024). Recent global burden and epidemiologic analyses indicate that ALD contributes substantially to liver-related mortality worldwide and remains a major driver of cirrhosis outcomes, underscoring the significant public health impact of ALD (Manikat et al., 2024; Wang et al., 2024). Research shows that heavy drinking over time results in clinically significant ALD in only 20%–30% of heavy drinkers, as susceptibility varies with individual genetic and health factors (Arab et al., 2022; Prince et al., 2023). The gut microbiome, composed of trillions of intestinal microorganisms, regulates human metabolism and immune function and is a critical factor in ALD (Wang et al., 2021). Scientists now consider it a vital regulator of ALD progression (Sarin et al., 2019).

The development of ALD results from multiple factors, including direct liver damage from prolonged ethanol use, metabolic disturbances, oxidative stress, and immune-mediated inflammation. The gut-liver axis is a crucial pathway in ALD development, describing how intestinal microbes and their products influence liver injury (Philips et al., 2022). The intestinal barrier is compromised by alcohol consumption, creating a “leaky gut” that allows bacteria and their products, including endotoxins, to enter the portal circulation and reach the liver (Maccioni et al., 2020). The entry of gut-derived microbial products into the liver via the portal circulation triggers hepatic Toll-like receptor activation and inflammatory responses, thereby intensifying hepatocellular damage and fibrosis (Wen et al., 2021). Chronic alcohol consumption alters the intestinal microbial community, resulting in dysbiosis. Research on ALD gut microbiota reveals that pathobionts, including *Enterobacteriaceae* and *Enterococcus faecalis* (*E. faecalis*), increase in abundance, while beneficial bacteria such as *Bifidobacterium* and *Faecalibacterium prausnitzii* decrease (Fairfield and Schnabl, 2021; Dukić et al., 2023)**.** The combination of gut barrier breakdown and microbial imbalance in the gut microbiota leads to increased liver damage in patients with ALD (Philips et al., 2022).

Research on ALD microbiomes has produced conflicting results; but scientists have not identified any specific microbial marker that distinguishes ALD (Grodin et al., 2024). The relationship between liver damage and microbiome changes in ALD remains unclear (Philips et al., 2022). It is not yet known whether microbiome alterations lead to liver disease or if they instead result from alcohol-related tissue destruction (Jung et al., 2022). The scientific community continues to debate how intestinal permeability affects disease progression in patients (Wang et al., 2021). The results of microbiome-based interventions during the early stages of treatment have been inconsistent (Grodin et al., 2024). Research on probiotic supplements for liver enzyme and inflammation management has shown limited success, as most studies have small sample sizes (Bernhardt et al., 2024; Xiong et al., 2024). The treatment of severe alcoholic hepatitis through fecal microbiota transplantation (FMT) shows promising results, but scientists need more research to confirm its safety and effectiveness (Pande et al., 2023; Taha et al., 2024). Bacteriophage therapy is an experimental treatment because scientists have not collected sufficient clinical data to support its use (Fujiki and Schnabl, 2023).

This review synthesizes current knowledge of ALD–microbiome interactions and evaluates emerging therapies targeting the gut–liver axis. We focused on literature from the last 5 years, identified via PubMed searches for “ALD microbiome,” and included key clinical trials and meta-analyses to ensure an evidence-based discussion. Ongoing debates about microbiome characteristics and treatment mechanisms are discussed, research gaps identified, and future directions proposed to guide the development of microbiome-based therapies for ALD.

## 2 Overview of the pathological mechanisms of ALD

The progression of ALD occurs when people drink heavily for extended periods because their liver function deteriorates from metabolic disturbances and inflammatory responses (Anouti et al., 2024). The following section describes how alcohol causes liver damage through three main processes: disrupted lipid metabolism, oxidative stress, and immune-mediated inflammation.

### 2.1 Disturbances in lipid and energy metabolism

The liver metabolizes ethanol primarily via alcohol dehydrogenase (ADH) and cytochrome P450 2E1 (CYP2E1), which together convert ethanol into acetaldehyde within hepatocytes. In addition, at high ethanol concentrations, the peroxisomal enzyme catalase oxidizes ethanol to acetaldehyde via an H_2_O_2_-dependent pathway (Contreras-Zentella et al., 2022). This catalase-mediated route complements the ADH and CYP2E1 pathways and contributes to oxidative stress during heavy alcohol exposure (Contreras-Zentella et al., 2022). As acetaldehyde accumulates, it forms adducts with proteins and DNA, leading to the generation of ROS, which damage mitochondria and trigger lipid peroxidation (Chen M. et al., 2023). Acetaldehyde also impairs mitochondrial respiratory chain function and shifts the cellular NAD^+^/NADH balance toward NADH. This redox imbalance, especially during chronic ethanol exposure, disrupts the tricarboxylic acid (TCA) cycle and exacerbates metabolic stress in hepatocytes (Yan et al., 2023).

Furthermore, ethanol skews hepatic lipid metabolism by inhibiting the activation of AMP-activated protein kinase (AMPK) and peroxisome proliferator-activated receptor (PPAR), thereby reducing fatty acid oxidation while promoting triglyceride synthesis (Namachivayam and Valsala Gopalakrishnan, 2021). Consequently, lipids accumulate in the liver, leading to fatty liver disease (hepatic steatosis), the earliest indicator of ALD. Beyond these direct hepatic effects, chronic alcohol misuse disrupts the gut microbiome, leading to an overgrowth of ethanol-producing bacteria (such as *Escherichia coli* (*E. coli*) and *Klebsiella pneumoniae*) that ferment dietary carbohydrates into ethanol (Park et al., 2021; Chen et al., 2022; Andaloro et al., 2025). The ethanol produced by these microbes enters the portal circulation and, under conditions of long-term alcohol exposure or gut dysbiosis, further increases the liver’s acetaldehyde burden and oxidative stress (Park et al., 2021). This additional ethanol load exacerbates hepatic metabolic disturbances and accelerates the progression of ALD.

### 2.2 Oxidative stress and mitochondrial damage

The development of ALD depends on the oxidative stress that occurs during alcohol consumption (Yan et al., 2023). The liver experiences elevated oxidative stress because CYP2E1-dependent ethanol metabolism generates substantial amounts of ROS, including superoxide anions and hydrogen peroxide. Damage to mitochondrial membrane lipids from ROS exposure leads to decreased mitochondrial membrane potential and reduced ATP production, disrupting cellular energy supply (Scarlata et al., 2024). The release of DNA fragments and other damage-associated molecular patterns (DAMPs) from damaged mitochondria triggers hepatic macrophage (Kupffer cell) activation, which in turn drives an inflammatory response through interleukin-1 (IL-1) release (Brahadeeswaran et al., 2023). The mitochondrial quality control system, which includes mitophagy and fusion–fission processes, becomes dysfunctional when prolonged cellular damage occurs due to alcohol exposure (Chen M. et al., 2023). Hepatocytes experience excessive mitochondrial protein acetylation when drinking heavily, which blocks fat breakdown, leading to fat accumulation and increased inflammation (Yan et al., 2023). The development of ALD depends on oxidative stress and mitochondrial damage, so researchers explore antioxidants and mitochondrial function improvement treatments as potential therapeutic approaches (Scarlata et al., 2024).

### 2.3 Immune, inflammatory, and fibrotic responses

The immune system produces two types of effects when alcohol consumption leads to inflammatory responses (Lotersztajn et al., 2022). The portal vein enables proinflammatory molecules from the gut to reach the liver, where they activate Kupffer cells via TLR4/NF-κB signaling, leading to the production of TNF and IL-1 cytokines (Wen et al., 2021). The release of pro-inflammatory cytokine**s** results in hepatocyte destruction and triggers hepatic stellate cell activation, which produces collagen and builds an extracellular matrix that leads to liver fibrosis (Chen M. et al., 2023). The production of anti-inflammatory factors, including IL-10 and interferon-α, decreases under alcohol consumption, creating an environment that promotes inflammation (Chen et al., 2024). The development of cirrhosis becomes possible when fibrosis progresses over time because of ongoing liver damage (Brahadeeswaran et al., 2023). The combination of metabolic, oxidative, and immune processes drives progression of ALD from steatosis to cirrhosis, with the gut microbiome influencing these pathogenic mechanisms (Yan et al., 2023).

## 3 Mechanisms of interaction between ALD and the gut microbiome

Research shows gut microbiota dysbiosis plays a major role in ALD development, but scientists need to determine its exact role in disease progression (Manzoor et al., 2022; Jew and Hsu, 2023). The gut microbiome of patients with ALD shows decreased microbial diversity, diminished beneficial bacteria, and excessive growth of harmful pathogens (Cabré et al., 2023). This section examines how prolonged alcohol consumption alters the gut microbiota, leading to liver dysfunction via intestinal barrier breakdown, changes in microbial metabolites, and immune system dysregulation (Jung et al., 2022).

### 3.1 Effects of alcohol on the gut microbiome

#### 3.1.1 Decreased microbial diversity and weakened colonization resistance

Chronic alcohol intake alters the gut environment – raising intestinal pH and destabilizing bile acid homeostasis – which disrupts the natural microbial balance (Sosnowski and Przybylkowski, 2024)**.** Human and animal studies show that chronic alcohol intake decreases beneficial bacteria (e.g., *Bifidobacterium* spp.) and creates conditions for harmful Enterobacteriaceae to overgrow (Cabré et al., 2023). The gut loses its protective function against external pathogens and opportunistic bacteria when its diversity is compromised (Manzoor et al., 2022; Shalaby et al., 2023). The essential bacterium *Akkermansia muciniphila* (*A. muciniphila*) decreases significantly in alcohol-fed mice, resulting in thinner intestinal mucus and compromised gut barrier function. The restoration of *A. muciniphila* populations in mice protected their livers from damage, demonstrating that this essential species plays a crucial role in preventing ALD progression (Grander et al., 2018).

#### 3.1.2 Damage to the intestinal mucosal barrier

The intestinal epithelial cells and the mucosal immune system are directly affected by alcohol, leading to gut barrier dysfunction (Cabré et al., 2023). Research demonstrates that moderate ethanol consumption leads to damage to intestinal tight junction proteins and to the blocking of stem cell proteins ISC1 and ZO-1, resulting in increased gut wall permeability (Elamin et al., 2012). Metabolites of alcohol, including acetaldehyde, disrupt tight junction proteins between epithelial cells, preventing epithelial cell regeneration (Kuo et al., 2022). The gut’s physical barrier becomes weaker because alcohol prevents normal epithelial cell replacement and tissue restoration (Cabré et al., 2023). Alcohol consumption decreases intestinal defenses by reducing mucus production and antimicrobial peptide secretion, including Reg3β and Reg3γ (Yue et al., 2023). Chronic alcohol consumption diminishes the production of IL-22 – a cytokine crucial for maintaining gut barrier strength. The addition of IL-22 to alcohol-consuming mice restores their gut health by increasing antimicrobial peptide production, enhancing gut barrier strength, and reducing endotoxin leakage and liver damage (Yue et al., 2023). The combined disruption of physical and immune barriers in the gut increases intestinal permeability, allowing bacteria and endotoxins to pass through the intestinal wall (Shibamoto et al., 2023).

#### 3.1.3 Expansion and translocation of proinflammatory microbiota

**FIGURE 1:**
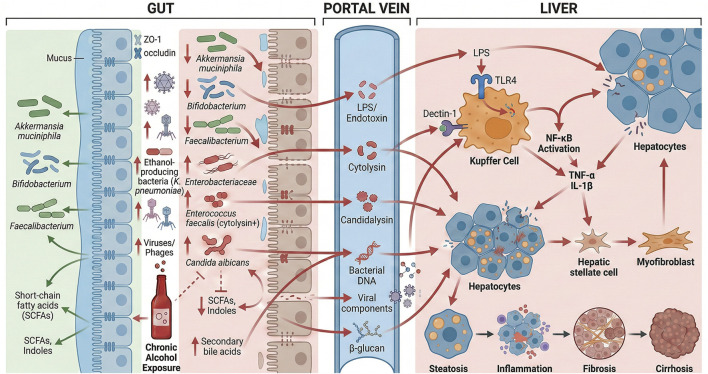
Disruption of the gut–liver axis in ALD through microbiota imbalance and barrier damage.

Alcohol-related dysbiosis leads to pathogen overgrowth and their spread outside the intestinal area (Juanola et al., 2024). The liver becomes exposed to virulence factors from *E. coli* when Gram-negative bacteria densely colonize the gut mucosa after chronic alcohol exposure (Yang et al., 2025). The liver is normally protected from these bacteria by an intact gut barrier. Research has discovered that *E. faecalis* strains producing the toxin cytolysin drive severe alcoholic hepatitis in patients. The bacteria *E. faecalis* exist in 80% of alcoholic hepatitis patients, and cytolysin-positive strains are present in 30% of these patients (Duan et al., 2019). In stark contrast, only about 10% of patients carrying cytolysin survive past the 6-month threshold, whereas approximately 96% of those without it do so (Duan et al., 2019). Toxic compounds from pathogenic gut bacteria cause liver damage when alcohol consumption disrupts the normal gut microbiome (Jew and Hsu, 2023). Bacteriophages targeting cytolytic *E. faecalis* strains in preclinical models successfully removed this pathobiont, resulting in complete prevention of ALD in treated mice (Duan et al., 2019)**.** Research shows that ALD develops through the destruction of beneficial gut bacteria and the growth of pathogenic bacteria that move from the gut to other body sites (Chen et al., 2022). The identification of these pathogens and their virulence factors enables healthcare professionals to develop new treatment approaches to stop the cycle of gut and liver damage (Fujiki and Schnabl, 2023). Figure 1 illustrates how chronic alcohol exposure disrupts the gut microbiome and compromises intestinal barrier integrity. In doing so, it promotes the portal translocation of microbial products to the liver, thereby driving hepatic inflammation and fibrogenesis.

#### 3.1.4 Gut virome alterations

Looking into ALD, most work has focused on shifts in gut bacteria (Jew and Hsu, 2023). Still, newer findings point toward a major role by the gut virome - viruses living inside the gut (Hsu et al., 2022; Jew and Hsu, 2023; One 2022 report identified distinct virome changes associated with specific ALD conditions, suggesting that viruses may help explain how the disease progresses (Hsu et al., 2022). A notable change in ALD is a dramatic increase in gut viral load, far above normal levels. This shift becomes even more pronounced in severe alcoholic hepatitis, where the gut microbiome becomes severely dysregulated (Jiang et al., 2020). The diversity levels among these groups show different patterns across age groups, which might result from antibiotic use and disease stage. The virome of ALD contains more eukaryotic viruses from the Parvoviridae and Herpesviridae families, and it has a greater number of bacteriophages targeting bacteria from the Enterobacteriaceae family and *E. coli* and *Enterococcus* species (Jiang et al., 2020). The viral lineages of *Staphylococcus* phages and herpesviruses have been linked to higher death rates and more severe illness in alcoholic hepatitis patients (Jiang et al., 2020). The models include specific viral taxa, allowing researchers to predict patient mortality over the next 90 days using hazard ratios as high as 35.8 (Jiang et al., 2020). In contrast, non-alcoholic fatty liver disease (NAFLD) shows reduced viral diversity (Lang et al., 2020a), underscoring alcohol’s unique effect on the virome. Alcohol, whether light or moderate, changes the viruses present in people with NAFLD when compared to those who do not drink at all (Hsu et al., 2023).

These changes imply that the virome actively shapes ALD pathogenesis via trans-kingdom interactions, rather than being a passive player in dysbiosis (Jew and Hsu, 2023). Bacteriophages, for instance, modulate the microbial ecosystem by lysing specific bacteria, altering the flora balance, and releasing molecules that worsen liver injury (Chen L. Y. et al., 2023). The depletion of beneficial commensals triggered by phages, or perhaps the proliferation of pathobionts, can foster the overgrowth of bacteria resistant to alcohol while ramping up endotoxin (LPS) release and compromising gut permeability (Chen L. Y. et al., 2023). In the setting of progressive ALD, links between specific phages and bacteria—such as Enterobacteria—tend to align with indicators of disease severity, including increased liver stiffness (Chen L. Y. et al., 2023). Moreover, phages could foster a “leaky gut” state by compromising the integrity of the mucosal barrier, thereby allowing the translocation of bacterial components and inflammatory mediators into the liver (Chen L. Y. et al., 2023). Notably, a considerable number of people dealing with alcohol use disorder (AUD) or ALD present with elevated gut permeability; take, for instance, the roughly 36%–40% of patients across certain ALD cohorts who demonstrate large-molecule translocation, thereby enabling microbial-associated molecular patterns (MAMPs) to travel to the liver through the portal vein (Maccioni et al., 2020). The enrichment of latent viruses, including Herpesviridae, shows that alcohol consumption weakens the immune system, leading to viral reactivation and elevated inflammation. For instance, Epstein-Barr virus positivity is linked to higher Model for End-Stage Liver Disease (MELD) scores in alcoholic hepatitis (Jiang et al., 2020). Thus, shifts in the virome amplify pro-inflammatory signals in ALD and extend the effects of bacterial dysbiosis. Additionally, studies underscore the interplay among the virome, bacteria, and fungi, in which pathogens such as *Streptococcus* and *Enterococcus* are often detected in ALD patient groups (Jew and Hsu, 2023).

Preclinical studies suggest a causal involvement of the virome in ALD, yet human data remain largely observational at this stage (Jiang et al., 2020; Hsu et al., 2022). The study has shown that bacteriophage therapy aimed at cytolysin-positive *E. faecalis* protects alcohol-fed mice from developing liver disease (Duan et al., 2019; Mendes et al., 2022). This treatment eliminated a pathogenic microbe and, in doing so, halted the inflammation and damage that arose from the gut (Duan et al., 2019). Notably, *Enterococcus* species are profoundly enriched in alcoholic hepatitis stool samples (5.59% vs. 0.023% in controls). Moreover, cytolytic *E. faecalis* is highly prevalent (about 80% of alcoholic hepatitis patients) and about 2,700-fold more abundant (by qPCR) in these patients, a finding that correlates with disease severity (Duan et al., 2019). Phage cocktails directed against this bacterium have been shown to reduce hepatic cytolysin levels (Duan et al., 2019; Mendes et al., 2022). At the same time, however, other observations suggest that virulent phages left uncontrolled could actually worsen ALD by helping sustain populations of harmful bacteria (Suh, 2021). Overall, the intestinal virome appears to act as a modulator of the gut-liver axis rather than a bystander (Wolstenholme et al., 2024).

Recent therapeutic strategies are increasingly focusing on the microbe-gut-liver axis (Alvarado-Tapias et al., 2025). Building on that, these interventions have made notable strides in recent studies. Take, for example, observations of increased viral diversity and a predominance of Enterobacteria and Lactococcus phages among patients with ALD (Hsu et al., 2022). Phage therapy, which precisely targets pathogenic bacteria, is supported by preclinical data indicating its clinical viability (Duan et al., 2019). In murine models, such treatments not only reduced bacterial burdens but also substantially mitigated markers of liver injury and fibrosis (Duan et al., 2019)—notably by targeting cytolysin-secreting *E. faecalis* in advanced ALD cases (Duan et al., 2019). Such approaches show promise for curbing inflammation through precise microbiome editing, effectively addressing issues such as cytolysin via virome-mediated bacterial control in ALD (Duan et al., 2019).

**FIGURE 2:**
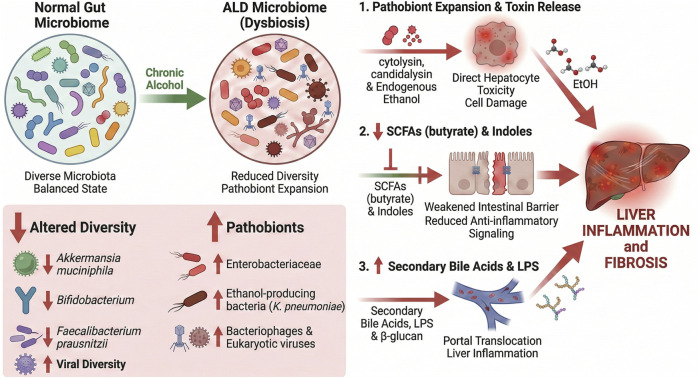
Gut dysbiosis in ALD leads to pathobiont expansion and loss of protective microbial metabolites.

Fecal virome transplantation (FVT) is a promising approach (Raeisi et al., 2023). By moving beneficial microbes from a donor into a recipient, it aims to transform their gut ecosystem - research links it to better results across various illnesses (Raeisi et al., 2023; Mao et al., 2024). For instance, in those dealing with AUD, abstaining for just 2 weeks sparked notable rises in helpful phages that zero in on *Propionibacterium*, *Lactobacillus*, and *Leuconostoc* species (Hsu et al., 2022). Drawing on metagenomic analyses, this form of virome plasticity appears to mitigate, to some extent, dysbiosis associated with ALD progression (Hsu et al., 2022). Preclinical studies have shown that FVT treatment reduces metabolic syndrome symptoms in mice by improving glucose tolerance when these animals eat high-fat diets (Mao et al., 2024). Moreover, FVT appears promising for addressing recurrent Clostridioides difficile infections in humans. From these early insights, small pilot studies suggest effective phage engraftment alongside notable cure rates through this method (Raeisi et al., 2023). Although FVT remains untested in ALD, it could prove to be a promising virome-focused strategy, one that might help rectify the phage imbalances commonly linked to AUD (Raeisi et al., 2023). In essence, the gut virome interacts with disrupted bacterial populations in ALD’s imbalanced intestinal environment, suggesting promising avenues for virome-focused diagnostic tools and therapies. As shown in Figure 2, alcohol-triggered gut dysbiosis involves a decline in microbial diversity, an increase in pathobionts, and a loss of beneficial microbial metabolites, all of which contribute to a compromised intestinal barrier that, in turn, drives subsequent liver damage.

### 3.2 The reciprocal impact of gut dysbiosis on the liver

The liver is affected by gut microbiome imbalances through multiple biological pathways, which could worsen ALD progression (Gao et al., 2024). The main mechanisms of liver damage include intestinal barrier failure (leading to endotoxin leakage), changes in microbial fermentation products, and immune system dysregulation driven by imbalances in the gut microbiota (Shibamoto et al., 2023).

#### 3.2.1 Gut fungal dysbiosis and immune activation in ALD

In addition to bacterial dysbiosis, chronic alcohol abuse is also associated with perturbations in the gut fungal community. The intestinal microbiome of patients with ALD shows reduced fungal diversity, making *Candida* species, including *Candida albicans* (*C. albicans*), the dominant microorganisms in their communities (Zeng et al., 2023). Such an imbalance in the mycobiome could undermine mucosal barriers, as alcohol-related damage to the gut lining allows fungal components such as β-glucan to enter the bloodstream (Zeng et al., 2023). Once fungal products enter the bloodstream, they trigger activation of the innate immune system. In the bulk of patients with ALD, elevated antifungal antibodies are a common finding, driven by elevated ASCA titers and raised serum β-glucan levels—clear signs of ongoing fungal translocation. Notably, higher ASCA levels in alcoholic hepatitis correlate with worse 90-day survival (Lang et al., 2020b). In particular, *Candida* overgrowth appears particularly pathogenic. The fungus *C. albicans* is usually a harmless commensal. Still, it turns into an invasive pathogen that produces virulence factors that make liver damage worse in people who drink alcohol. Candidalysin functions as a virulence factor that research shows damages hepatocytes while increasing inflammation (Chu et al., 2020). Strains of *C. albicans* that produce candidalysin markedly aggravate ethanol-triggered liver damage in mice, and detecting this toxin in patients’ stool samples is closely associated with a poorer prognosis (Zeng et al., 2023).

Fungal dysbiosis may likewise disrupt the adaptive immune response. Consider, for example, how β-glucan engages receptors such as Dectin-1 on Kupffer cells, prompting IL-1β secretion and activating inflammatory pathways that tilt the balance toward Th17 polarization. Research indicates that prolonged ethanol exposure drives the proliferation of Th17 cells targeted to *C. albicans* in the gut and bloodstream, and these cells might subsequently relocate to the liver to release IL-17, thereby potentially intensifying inflammation (Zeng et al., 2023). These Th17 responses are associated with neutrophil recruitment and liver damage. Moreover, they may undermine regulatory mechanisms. For example, dendritic cells sensing β-glucan can convert regulatory T cells (Tregs) into IL-17 producers, suggesting mycobiome-induced Treg dysfunction (Zeng et al., 2023).

Research suggests that cross-kingdom microbial interactions may amplify pathological effects in ALD. Indeed, fungal overgrowth often coincides with bacterial and viral dysbiosis (Chu et al., 2020; Jew and Hsu, 2023). These disruptions appear capable of synergistically increasing gut permeability and inflammation through the gut-liver axis. In clinical practice, an altered mycobiome correlates with disease severity and fibrosis stage (Viebahn et al., 2024). Research studies conducted before clinical trials have demonstrated that non-absorbable antifungal medications, such as nystatin, can effectively reduce *C. albicans*-specific Th17 immune responses while controlling fungal growth and decreasing IL-1β-induced Th17 inflammation and liver damage (Zeng et al., 2023). The treatment of severe human ALD with FMT shows promise for improving survival rates in steroid-resistant patients during the first 3 months of treatment (Shasthry, 2020). Gut fungi influence the progression of ALD by activating Th17 cells and potentially interfering with immune system regulation (Zeng et al., 2023). This underscores the mycobiome’s role as a key component of the gut-liver axis (Viebahn et al., 2024).

#### 3.2.2 Disruption of microbial metabolites

The gut microbiome produces beneficial metabolites that help maintain equilibrium between the gut and liver systems when it is functioning properly (Hsu and Schnabl, 2023; Yang and Schnabl, 2024). The development of dysbiosis through alcohol consumption disrupts microbial metabolite production (Ganesan and Suk, 2022). Reducing short-chain fatty acid (SCFA)-producing bacteria decreases beneficial SCFA levels in intestinal tissues. The reduction of SCFAs, which serve as essential nutrients for colonocytes and as regulators of immune homeostasis, leads to intestinal barrier weakness, allowing more endotoxins to enter the liver and increasing inflammation (Shalaby et al., 2023). The liver develops increased inflammation as a result of this process (Zhang et al., 2025). Dysbiosis contributes to the excessive production of specific microbial metabolites, leading to adverse effects (Jung et al., 2022). The liver experiences problems with microbial bile acid processing because extended alcohol consumption alters the microbiome (Yan et al., 2023). Alcohol-induced microbiome changes result in higher production of deoxycholic acid and specific secondary bile acids, which activate inflammatory pathways and worsen liver damage (Fairfield and Schnabl, 2021; Zhu et al., 2023). The severity of alcoholic hepatitis in patients directly correlates with their stool secondary bile acid levels because of higher 7α-dehydroxylating bacterial activity (Muthiah et al., 2022). Beneficial tryptophan-derived indole metabolites, which have anti-inflammatory properties, become less abundant (Mrdjen et al., 2023). The liver is injured in ALD because the gut microbiome disrupts its natural metabolite balance, resulting in decreased protective compounds (SCFAs and indoles) and increased toxic bile acids (Ganesan et al., 2024).

#### 3.2.3 Immune imbalance and inflammation amplification

**FIGURE 3:**
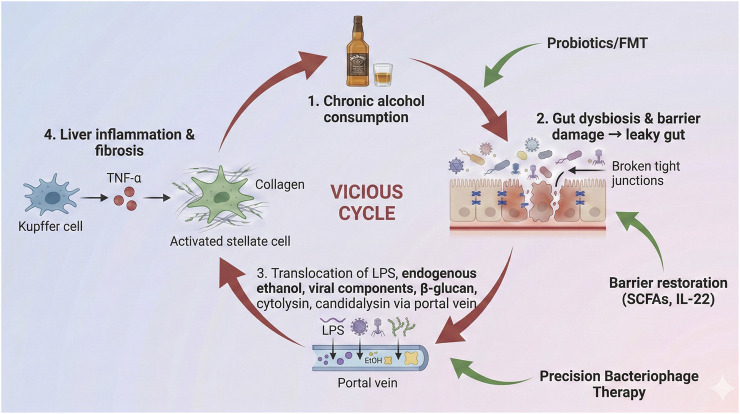
Self-reinforcing cycle of dysbiosis, barrier dysfunction, and liver inflammation in ALD.

The immune system remains persistently active due to prolonged dysbiosis in ALD (Zeng et al., 2023). The leaky gut condition allows immune cells in the liver to detect microbial products (e.g., LPS), which triggers persistent Toll-like receptor 4 (TLR4) and NOD2 signaling and produces elevated levels of inflammatory cytokines TNF-α and IL-1β (Shalaby et al., 2023). The persistent activation of immune cells exceeds the body’s ability to mount anti-inflammatory responses, leading to a proinflammatory immune state (Lotersztajn et al., 2022). Excessive growth of *Candida* species alters the immune system, driving Th17-mediated responses (Zeng et al., 2023). The absence of immunoregulatory microbial metabolites, including butyrate (an SCFA), disrupts the normal development of regulatory T-cells and Kupffer cell tolerance (Zhang et al., 2024). The liver undergoes continuous damage and fibrosis due to an excessive inflammatory response (Zhang et al., 2025). The immune system becomes unbalanced due to alcohol-related gut dysbiosis, which produces heightened liver inflammation and fibrosis (Lotersztajn et al., 2022). Figure 3 summarizes the self-amplifying gut–liver inflammatory loop in ALD and highlights potential intervention points along this pathway.

#### 3.2.4 Gut dysbiosis in the progression and complications of late-stage ALD

In decompensated cirrhosis (late-stage ALD), profound gut dysbiosis occurs and the intestinal barrier function is severely compromised. These changes are associated with worsening liver disease and serious complications (Jew and Hsu, 2023; Grodin et al., 2024). A heightened permeability of the intestinal barrier facilitates the translocation of bacteria and the broader circulation of microbial components, such as lipopolysaccharide (LPS), which, in turn, escalates liver inflammation and hastens the shift toward clinical decompensation (Jew and Hsu, 2023; Grodin et al., 2024). Progressive cirrhosis is associated with loss of beneficial commensal bacteria and an overgrowth of pathogenic bacteria (e.g., Enterobacteriaceae). These changes correlate with disease severity and adverse clinical outcomes (Bajaj et al., 2014; Grodin et al., 2024).

Beyond sustaining inflammation, gut dysbiosis plays a key part in driving hepatic fibrogenesis via several overlapping pathways. In particular, the decline in beneficial microbial activities, such as the generation of SCFAs, undermines the strength of the epithelial barrier and disrupts signals that curb inflammation, allowing the ongoing passage of pathogen-associated molecular patterns, including LPS, into the system. These signals, in turn, engage Kupffer cells via TLR4, triggering pro-inflammatory and profibrogenic pathways that ultimately lead to hepatic stellate cell activation and collagen deposition (Chang et al., 2025). Dysbiosis also disrupts bile acid–FXR signaling, especially under cholestatic conditions, undermining mucosal defenses and promoting bile acid profiles that are hepatotoxic (Chang et al., 2025). Alongside this, certain microbial byproducts, trimethylamine N-oxide (TMAO) among them, show heightened levels in severe alcohol-associated liver conditions—and intriguingly, blocking TMAO’s precursor in lab settings has proven effective at easing alcohol-driven liver damage in animal studies (Alvarado-Tapias et al., 2025). Taken together, these mechanisms foster a vicious cycle within the gut-liver axis, driving fibrosis forward and hastening the slide into cirrhosis.

These repercussions from dysbiosis-mediated gut-liver axis disruption stand out most starkly in cirrhosis’s decompensated phase, where they present as grave complications—such as spontaneous bacterial peritonitis (SBP) or hepatic encephalopathy (HE), both of which are potentially lethal complications (Chang et al., 2025). SBP typically develops when bacteria originating from the gut cross a weakened intestinal barrier and enter the ascitic fluid. This translocation is typically characterized by Gram-negative Enterobacteriaceae, a process further aggravated by dysbiosis and compromised immunity that often accompany cirrhosis (Höppner et al., 2023). Similarly, hepatic encephalopathy arises from dysbiosis along the gut-liver-brain axis. In cases of decompensated cirrhosis, compromised liver detoxification coupled with portosystemic shunting leads to the buildup of neurotoxins—ammonia stands out here, generated by urease-producing bacteria in the gut. Meanwhile, endotoxemia associated with dysbiosis, coupled with broader systemic inflammation, tends to intensify neuroinflammation (Bajaj et al., 2014). Patients with HE routinely show a distinctive microbiome pattern, one enriched in Proteobacteria while short on beneficial taxa. From this angle, the resulting dysbiosis closely aligns with hyperammonemia, elevated inflammatory markers, and cognitive deficits (Chang et al., 2025). It's worth noting that these complications are closely intertwined; for example, an infection like SBP can often trigger HE episodes, underscoring the pivotal role of gut-derived inflammation in advanced ALD.

Such insights hold substantial therapeutic promise, prompting a growing focus on interventions targeting the microbiome. Lactulose, as a prebiotic, and rifaximin, which curbs bacteria responsible for urease and endotoxin production, remain pivotal treatments for managing HE, highlighting the practical effectiveness of influencing gut microbiota dynamics (Zacharias et al., 2023). Beyond these conventional approaches, FMT appears promising based on initial clinical trials in alcohol-related cirrhosis, where it enhances gut microbial diversity and mitigates complications such as hepatic encephalopathy more effectively than routine treatments (Ichim et al., 2025). Nevertheless, one must recognize that the bulk of mechanistic connections between gut dysbiosis and the fibrogenic advancement in ALD stem largely from preclinical models or correlative human investigations. From this standpoint, firm causal associations remain to be confirmed (Cheng et al., 2025). Most therapies targeting the microbiome today mainly address complications, not the core disease mechanisms themselves—yet rebalancing gut-liver interactions could, in turn, affect how fibrosis advances. From this viewpoint, such a disease-altering possibility remains largely speculative and calls for solid evidence from prospective, long-term, and in-depth mechanistic investigations (Shen Y. et al., 2025).

### 3.3 Host genetics and microbiome interaction mechanisms

Host genetic factors are pivotal in modulating the risk and progression of ALD. Several genetic variants have been identified as significant contributors to ALD severity, including *PNPLA3*, *TM6SF2*, *MBOAT7*, and *HSD17B13* (Wang et al., 2025). For example, the *PNPLA3* rs738409 (I148M) variant leads to a two-to three-fold higher risk of developing alcoholic cirrhosis among heavy drinkers (Galvanin et al., 2025). Genome-wide analyses have discovered that *TM6SF2* and *MBOAT7* variants with dysfunctional properties lead to cirrhosis development in people who consume alcohol (Choudhary and Duseja, 2021). In contrast, the *HSD17B13* rs72613567: TA variant protects against severe liver fibrosis by reducing risk by 20%–50% (Demirtas and Yilmaz, 2024). The combination of multiple risk alleles in *PNPLA3* and *TM6SF2* genes leads to a substantial increase in cirrhosis risk for individuals who carry these variants (Shankarappa et al., 2023).

Genetic variations between individuals influence liver function by affecting lipid metabolism and immune system responses that regulate the gut-liver axis. The *PNPLA3* and *TM6SF2* genes regulate liver fat storage and metabolism, thereby influencing bile acid production levels that modulate gut microbiota structure (Hsu and Schnabl, 2023). Genetic variations in immune-related genes, such as *TLR4* and *NOD2*, influence how the liver responds to microbial signals via inflammation, establishing a connection between genetic factors and microbiome-related processes (Shen H. et al., 2025). Research suggests that the host’s genetic makeup shapes the composition of the gut microbiota. Genetic factors associated with immune regulation or metabolism can influence gut microbiota composition (Miteva et al., 2023). Research on alcohol-dependent patients reveals that people carrying high-risk variants of *PNPLA3* and *TM6SF2* develop gut microbiota profiles containing more inflammatory bacteria (Vishnubhotla et al., 2022). Studies indicate that heavy drinkers with advanced ALD have higher levels of *Proteobacteria* and lower levels of *Faecalibacterium spp*. (Kuo et al., 2025). The combination of genetic variations with microbial populations leads to increased host inflammation, resulting in disease progression (Dukić et al., 2023).

Moreover, differences in immunity-related genes, such as *TLR4* (and, to a lesser extent, *NOD2*), can influence how the host responds to gut-derived compounds, such as lipopolysaccharides (LPS) (Shen H. et al., 2025). People with certain genetic variants that are less reactive to microbial imbalances might have milder immune responses, leading to reduced inflammation after drinking alcohol. The severity of inflammation increases when individuals have hyper-responsive genetic variants (Tang et al., 2023). The fibrosis-associated gene *TM6SF2* regulates gut permeability and lipid metabolism, producing secondary effects on microbial communities (Choudhary and Duseja, 2021).

Research has identified a protective *HSD17B13* variant that shows promise in decreasing the occurrence of alcoholic hepatitis (Wang et al., 2025). This specific variant controls lipid metabolism by decreasing liver fat storage, linking it to gut-liver axis stability and immune regulation (Demirtas and Yilmaz, 2024). Integrating genetic data with microbiome research will become essential for understanding the different manifestations of ALD and identifying high-risk patients who need targeted treatments (Chetty and Blekhman, 2024).

In summary, a patient’s genetic profile determines their inherent susceptibility to ALD (Wang et al., 2025). The gut microbiome interacts with genetic factors to either accelerate or slow the progression of the disease (Li et al., 2024). Developing individualized ALD treatment plans requires studying how genetic factors interact with microbial populations (Listopad et al., 2024).

### 3.4 Advances in multi-omics integration research

Modern biomedical research increasingly employs multi-omics integration strategies to explore complex diseases (Hieromnimon et al., 2025). The combination of metagenomics with metabolomics, transcriptomics, and host genomics enables researchers to study the complete interactions between hosts and their microbiomes in the context of ALD (Hyun et al., 2022). The metagenomic approach allows researchers to examine how the gut microbiota functions in ALD patients (Jung et al., 2022). Analysis of blood and fecal samples through metabolomics helps scientists detect particular microbial compounds that correlate with disease severity or progression (Wang et al., 2022). The combination of these datasets shows how microbial genes interact with metabolite patterns and liver gene expression. Research using 16S rRNA gene sequencing combined with metabolomics and lipidomics in animal models of ALD shows that an imbalance between the *Clostridium* and *Romboutsia* genera leads to liver damage through PPARα suppression, lipid accumulation, and changes in metabolites, including 4-hydroxyphenylacetic acid (Zhang T. et al., 2023). This research demonstrates that multi-omics approaches help scientists understand how gut bacteria influence host metabolic networks. Research using single-cell RNA sequencing (scRNA-seq) has shown its value in studying the liver microenvironment in ALD, revealing how microbial factors affect immune responses and cellular diversity (Cao et al., 2023). Research shows that Kupffer cells and other hepatic cell types respond differently to LPS (a microbial byproduct), activating distinct immune pathways. The discovery of multiple macrophage subsets and their inflammatory roles in ALD challenges the previous belief that alcohol-induced dysbiosis triggers uniform immune cell activation (Ren et al., 2023; Sun et al., 2023). Identifying specific vulnerabilities in these cell populations enables the development of targeted treatments that control immune reactions in ALD patients (Atif et al., 2022; Sun et al., 2023). The combination of advanced methods with metagenomic data and other omics data enables a better understanding of fundamental ALD processes and creates opportunities to develop novel diagnostic tools and treatments. The analysis of multi-omics datasets faces major technical challenges because distinguishing genuine biological markers from experimental errors is difficult (Das et al., 2022; Ghosh et al., 2025a).

#### 3.4.1 Integrative biomarker discovery

Scientists are now studying multiple data types together to create composite biomarkers that provide better ALD progression prediction than individual factors alone (Niu et al., 2022). The combination of gut microbiome patterns with host genetic variations correlates with severe liver fibrosis development in heavy drinkers with ALD (Miteva et al., 2023). The combination of gut microbiota metabolites with cytokine patterns shows promise for predicting short-term outcomes in patients with alcoholic hepatitis (Im, 2023). Machine learning models of plasma multi-omics data reveal patterns linking *Clostridium* species and *Bacteroides fragilis* to elevated urobilinogen levels and changes in neutrophil gene expression (including IL-8, TNF-α, and NOX upregulation) that predict poor response to corticosteroids in severe alcoholic hepatitis (Maras et al., 2023). The development of multi-omics tests that combine microbial DNA with host RNA markers in blood will enable precise patient risk assessment for ALD and allow doctors to choose individualized treatments (Calzadilla et al., 2024). The integration of multiple data types helps scientists identify distinct disease subtypes—for example, distinguishing patients with gut-driven inflammation from those with metabolic damage—by combining omics patterns that guide specific treatment approaches (Gao et al., 2021).

#### 3.4.2 Personalized therapeutic target identification

The analysis of multi-omics datasets enables doctors to identify personalized treatment options for patients. The combination of microbiome, metabolome, and genome data allows researchers to pinpoint which specific patient pathways need intervention (Gao et al., 2021; Ganesan and Suk, 2022). The multi-omics profile of an ALD patient might show elevated microbial ethanol production and the absence of butyrate-producing bacteria (Chen et al., 2022), which can be treated with personalized synbiotic therapy (Jung et al., 2022). The treatment approach for patients with endotoxemia and hyperinflammatory gene expression should include TLR4 inhibition or antibiotic/probiotic therapy to decrease endotoxin-producing bacteria (Tang et al., 2023). Researchers performed an integrated study of gut microbiota and liver transcriptomics to discover *Enterococcus* toxin as a new bacterial target. Using a custom bacteriophage, they targeted this toxin in mice, stopping the disease from advancing (Duan et al., 2019). The integration of multi-omics data with individualized treatment approaches represents the current state of precision medicine for ALD patients (Listopad et al., 2024).

#### 3.4.3 Challenges and potential of multi-omics integration

Implementing multi-omics methodologies for ALD research poses several challenges, including complex data integration requirements and substantial computational demands. Combining multiple omics datasets requires powerful bioinformatics software to detect direct relationships between diverse biological elements (Zhou et al., 2025). Analyzing large datasets with numerous variables is challenging when working with small patient groups, leading to potential overfitting and false correlations (Listopad et al., 2024). Gathering multiple types of biological data using multi-omics methods often requires invasive procedures (e.g., liver biopsies for transcriptomics) and expensive testing, making it difficult to study large cohorts. Comparing and combining different studies requires standardized data processing and sharing methods to achieve meaningful results (Thiele et al., 2024). Despite these obstacles, the potential benefits of multi-omics remain substantial. Forming research consortia enables the collection of extensive multi-omics data from ALD patients, allowing monitoring of molecular changes in the gut–liver system throughout treatment and disease progression (Kuo et al., 2025)**.** Researchers are developing new analytical methods that combine network-based approaches with causal inference techniques to extract functional information from multi-omics data (for example, identifying microbial metabolites that link gut bacterial genes to host signaling pathways) (Ghosh et al., 2025a). These multi-omics insights may yield novel therapeutic targets (e.g., specific microbial enzymes or host pathways) and guide the development of microbiome-based drugs or precision probiotics. Through technological advancements and collaborative efforts, multi-omics integration will usher in a new era of ALD research, enabling individualized medical treatment.

## 4 Gut microbiome-related biomarkers

Research into the gut-liver axis of ALD has led scientists to focus on identifying microbiome-based biomarkers to help doctors diagnose ALD, predict its progression, and assess treatment effectiveness (Im, 2023). The biomarkers for ALD include specific microbial species, their metabolites, and host compounds that are linked to ALD severity and patient outcomes. The three main categories of these markers include microbial signatures, metabolomic indicators, and immune-inflammatory signals, which demonstrate the continuous interaction between the gut and liver during ALD development.

### 4.1 Microbial signatures

High-throughput 16S rRNA sequencing combined with metagenomic analyses has revealed distinct microbial patterns in patients with ALD (Park et al., 2024). The gut microbial diversity of patients with alcoholic cirrhosis shows lower alpha-diversity than that of healthy individuals (Li et al., 2024). The phylum Proteobacteria (e.g., Enterobacteriaceae) is increased beyond normal levels, while Firmicutes show decreased numbers of *Faecalibacterium prausnitzii* and Clostridiales, according to multiple studies on severe ALD (Li et al., 2025). Intestinal fungal dysbiosis, particularly overgrowth of *Candida* species including C. albicans, contributes to the development and progression of ALD (Viebahn et al., 2024). Elevated C. albicans abundance is associated with more severe ALD stages such as cirrhosis, and fungal immune responses (e.g., elevated ASCA) correlate with higher mortality in alcoholic cirrhosis (Lang et al., 2020b). Cytolysin-producing *E. faecalis* represents a separate bacterial risk marker for severe alcoholic hepatitis and mortality, supported by independent studies (Yang et al., 2017; Duan et al., 2019; Lang et al., 2020b). The analysis of stool microbiomes shows promise for developing non-invasive methods to monitor disease progression in clinical settings (Shen Y. et al., 2025). The combination of high *Enterococcus* abundance with the presence of the cytolysin gene would indicate a patient faces a higher risk of poor outcomes (Cabré et al., 2023). Microbiome composition shows predictable patterns during treatment, including the restoration of *Lactobacillus* during abstinence and therapy periods (Grander et al., 2018). Scientists are developing composite indices, such as the “dysbiosis index,” to quantify deviations in the microbiome from a healthy state (Bajaj et al., 2014). The dysbiosis index in ALD patients shows a direct correlation with liver disease severity and the number of complications (Bajaj et al., 2014). The identified microbial patterns help scientists understand disease mechanisms and have potential as clinical biomarkers once reliable stool-based tests are developed (Yu et al., 2025).

### 4.2 Microbial metabolites as biomarkers

The gut-liver axis allows microbiota-derived metabolites to cross into the circulation, and scientists now use these compounds as functional biomarkers to study ALD. Examples include SCFAs, bile acids, and aromatic compounds (like phenylacetylglutamine) produced by gut microbes. ALD progression correlates with decreased fecal levels of butyrate and other SCFAs, as the gut contains fewer bacteria that produce these compounds (Calzadilla et al., 2024). Serum levels of secondary bile acids, including deoxycholic acid (DCA) and lithocholic acid (LCA), increase in ALD patients as their microbiota changes bile acid composition (Calzadilla et al., 2024). These compounds show potential as biomarkers for assessing disease progression and liver damage associated with cholestasis (Calzadilla et al., 2024). Moreover, in certain chronic drinkers, the ethanol generated by intestinal microbes might further impair liver function, thereby aggravating their overall health. Researchers have been exploring markers associated with microbial ethanol production, aiming to shed light on the persistent, self-reinforcing cycle of ALD among patients (Chen et al., 2022; Ferrandino et al., 2024; Andaloro et al., 2025). Tryptophan metabolites like indole-3-acetic acid and indolepropionic acid — normally produced by a healthy microbiome and providing anti-inflammatory effects in the gut-liver axis, — are found at reduced concentrations in ALD. Their deficiency could serve as a marker of dysbiosis severity (Mrdjen et al., 2023). Advanced metabolomic analyses have revealed new potential biomarkers, including 2-butanol as a microbial fermentation product that shows preliminary links to alcoholic hepatitis (Ferrandino et al., 2024). These metabolites function both as biomarkers of dysbiosis and as contributors to disease progression, making them suitable for therapeutic monitoring. For example, an increase in butyrate (an SCFA) through an intervention could correspond with improved patient outcomes, since butyrate is a beneficial compound (Calzadilla et al., 2024).

### 4.3 Host immune biomarkers

The host immune system reacts to microbial antigens present during gut dysbiosis by producing immune molecules that serve as biomarkers of microbiome alterations. Plasma levels of LPS-binding protein (LBP) and soluble CD14 (sCD14) increase in ALD patients to levels that match disease severity, as these molecules indicate gut-derived endotoxin exposure (Maccioni et al., 2020). Blood levels of intestinal fatty acid–binding protein (I-FABP) increase in ALD patients, indicating their enterocytes have been damaged due to gut barrier breakdown caused by dysbiosis (Hansen et al., 2025). The adaptive immune system produces endotoxin-specific antibodies (EndoCAb) and *Candida*-specific antibodies at higher levels in ALD patients, suggesting greater microbial translocation (Zeng and Schnabl, 2024). Research supports using a panel that includes high LBP levels, low EndoCAb concentrations, and elevated antifungal antibodies to predict alcoholic hepatitis risk, though no specific validated panel exists yet (Maccioni et al., 2020). Similar combinations are being explored for prognostic purposes in ALD and related conditions (e.g., primary sclerosing cholangitis) (Zeng and Schnabl, 2024). Blood tests measuring circulating bacterial DNA or 16S rRNA via polymerase chain reaction (PCR) enable doctors to directly detect microbial translocation, which has been linked to outcomes in severe ALD cases (Li et al., 2024; Haedge et al., 2025). Measuring these host-response biomarkers in blood provides greater clinical accessibility than fecal tests, as they can be detected through routine blood sampling. These biomarkers indirectly reflect gut health; for instance, reductions in plasma endotoxin and sCD14 levels in ALD patients during treatment indicate improvements in gut barrier function and a correction of microbial imbalance (Wang et al., 2021; Haedge et al., 2025).

### 4.4 Multi-omics and composite biomarker indices

The complex nature of ALD requires biomarkers that combine different “omic” data layers for maximal insight. Researchers are developing unified scores that integrate microbiome data with clinical indicators to understand disease progression better. Research indicates that combining *E. faecalis* abundance with serum LPS levels and systemic inflammation markers (such as IL-8) yields a biomarker that predicts 90-day survival in patients with alcoholic hepatitis. Multi-omics analysis (Im, 2023) shows that this method yields better predictive performance than Maddrey’s discriminant function. The development of machine learning models for multi-omics data analysis enables researchers to create a “microbiome health score” system that predicts disease progression in ALD patients. The algorithm generates disease progression probabilities by analyzing stool metagenomics data, serum metabolomics results, and standard laboratory tests (Ghosh et al., 2025a). An AI-based biomarker system in a clinical study used microbial and metabolite patterns to predict which alcoholic hepatitis patients would benefit from steroid treatment (Dunn et al., 2024). Researchers now focus on validating these complex biomarkers across different research groups while developing practical medical tests based on them (Im, 2023). For instance, rather than sequencing a whole metagenome, key indicator species (such as a high *Enterococcus*-to-*Lactobacillus* ratio) might be measured via targeted PCR as a proxy for the broader microbial signature. The goal is to develop practical biomarkers that will help doctors detect ALD early, forecast disease progression, and track patient responses to microbiome-based treatments, including probiotics and FMT (Lang et al., 2020c).

## 5 Microbiome-targeted therapeutic strategies in ALD

Traditional management of ALD (e.g., alcohol abstinence, nutritional support, and corticosteroids) is increasingly being complemented by strategies that target the gut microbiome (Anouti et al., 2024). In recent years, researchers have explored several microbiome-targeted approaches for ALD, including probiotics, prebiotics, FMT, bacteriophage therapy, and postbiotics. Each of these interventions aims to restore a healthier gut microbiome or eliminate dysbiotic triggers of liver injury. This review examines the current research on such microbiome-focused treatments, evaluating their effectiveness, biological mechanisms, and potential drawbacks.

### 5.1 Probiotics and prebiotics

The scientific community is increasingly interested in probiotic and prebiotic approaches for ALD treatment because these methods enable the restoration of gut microbiota equilibrium, improve intestinal barrier function, and modulate immune responses (Xiong et al., 2024). Studies in animal models and clinical trials indicate that certain probiotics and prebiotics can benefit ALD patients (Xiong et al., 2024). In particular, three probiotic strains (from genera *Lactobacillus*, *Bifidobacterium*, and *Enterococcus*) and prebiotic fibers (inulin-type fructans and oligosaccharides) have shown positive effects on liver function and inflammation. Kim et al. (2022) demonstrated that ethanol-fed mice showed improved liver health after receiving *Lactiplantibacillus plantarum* J266 and *Lacticaseibacillus rhamnosus* GG (LGG). These probiotics protected the mice’s livers from alcohol-induced damage, as evidenced by improved serum alanine aminotransferase (ALT) and aspartate aminotransferase (AST) levels and reduced hepatic IL-6 and fat accumulation (Kim et al., 2022).

In human studies, the findings have varied to some extent, though they have been generally positive overall (Xiong et al., 2024). Take, for instance, a randomized trial among patients with moderate alcoholic hepatitis, where treatment using LGG correlated with improved liver function indicators following a month of intervention (Vatsalya et al., 2023)—notably, this included a marked decrease in the AST: ALT ratio along with a reduced MELD score. However, that study did not evaluate longer-term outcomes (e.g., 6-month survival). More broadly, Xiong and colleagues (2024) conducted a meta-analysis of 9 RCTs, which showed that probiotic therapy led to modest improvements in liver enzymes (average ALT reduction of 13 U/L, AST reduction of 17 U/L) and a slight increase in albumin. Notably, there was no significant change in key pro-inflammatory cytokines (e.g., TNF-α, IL-6), suggesting that probiotics primarily improve gut barrier function (reducing endotoxin leakage) rather than directly dampening systemic inflammation (Xiong et al., 2024). Taken together, the findings suggest that probiotics and prebiotics might help reduce liver damage in ALD, presumably by bolstering the gut-liver axis and reducing the inflammatory burden. However, their impact is limited. Furthermore, high heterogeneity among studies (e.g., differences in trial design, specific microbial strains/doses, and patient baseline microbiomes) complicates generalizing the results (Kim et al., 2022; Xiong et al., 2024).

Recent analyses show that probiotic or prebiotic therapy provides only modest improvements in liver enzymes and no significant antifibrotic effect in ALD (Xiong et al., 2024)**.** In light of heterogeneous trial results, the overall evidence for probiotics/prebiotics yielding substantial clinical benefit remains weak (Xiong et al., 2024). Research into prebiotics has produced findings similar to those of the parallel studies. The pilot RCT of inulin during alcohol withdrawal showed that participants who received inulin developed distinct gut microbiota patterns, including higher *Bifidobacterium* levels, but their AST and ALT values increased over the 3-week study period (Amadieu et al., 2022). The study results showed that inulin supplementation failed to enhance liver function, although it increased SCFA production in the research participants (Amadieu et al., 2022). Thus, current evidence for a prebiotic benefit in ALD is weak. In summary, short-term probiotic or prebiotic treatments yield only modest improvements in gut barrier function and liver enzyme levels in patients with ALD (Amadieu et al., 2022). Still, their therapeutic benefits have not been definitively established. Larger, well-controlled RCTs are needed to determine whether specific fibers or synbiotic combinations can meaningfully protect the alcohol-injured liver.

The treatment results from probiotic or prebiotic therapy show significant differences between individual patients. The effectiveness of a given probiotic strain likely depends on host factors (genetics, diet, baseline microbiome). Notably, probiotics and prebiotics have a good safety profile with very few serious adverse events reported, and they are relatively low-cost interventions (Bernhardt et al., 2024). However, larger multi-center trials are needed to identify optimal strains, doses, and treatment durations, and to confirm any clinical benefits (Xiong et al., 2024).

**FIGURE 4:**
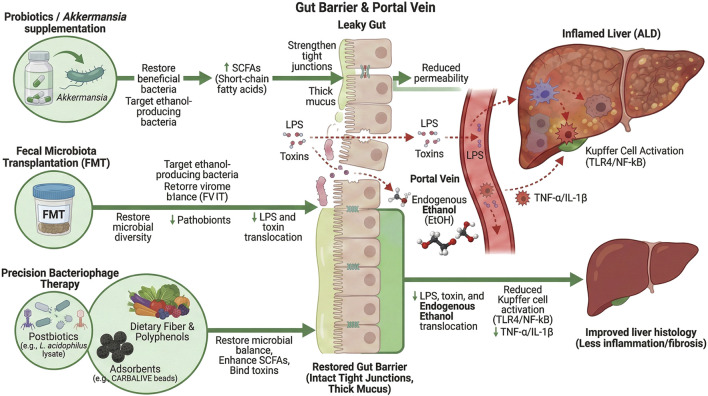
Microbiome-based therapeutic strategies restore gut barrier integrity and reduce liver inflammation in ALD.

Synbiotic therapies (combining probiotics with prebiotics) may offer enhanced benefits via multiple mechanisms: strengthening intestinal tight junctions, modulating immune responses, and generating beneficial metabolites like SCFAs (Bernhardt et al., 2024). In practice, synbiotic treatment in chronic ALD has shown only modest changes in gut microbiota composition (Jung et al., 2022). Accordingly, research is expanding to novel microbiome-based strategies–such as personalized bacteriophage cocktails and engineered probiotics–aiming for greater correction of dysbiosis and reduction of ALD-associated inflammation. Figure 4 illustrates how microbiome-directed therapies can restore the gut barrier (reducing the translocation of bacteria and toxins) and thereby lessen liver inflammation and slow fibrosis progression.

### 5.2 Fecal microbiota transplantation (FMT)

FMT involves using processed donor stool to restore gut microbiota balance through three possible delivery methods: colonoscopy, nasoduodenal tube, and capsule administration. FMT transfers an entire community of gut microorganisms from a healthy donor to a patient, and is an established, highly effective treatment for recurrent *Clostridioides difficile* infection, leading to full microbiome restoration in that context (Ma L. et al., 2025). Given this success, FMT is now being explored in ALD. Early pilot studies have suggested it can be performed safely in alcoholic hepatitis patients (Sharma et al., 2022), but further research is needed to confirm its efficacy in improving ALD outcomes. Recent meta-analyses indicate that FMT in ALD has no significant safety signals (no serious adverse effects reported) and may substantially improve short-term outcomes (Pakuwal et al., 2025). For example, pooled trial data in severe alcoholic hepatitis showed that FMT was associated with improved 28-day survival without procedure-related complications. Despite FMT’s invasive nature, it is being actively investigated as a means to rebalance the gut microbiome in ALD patients.

Multiple studies now confirm that FMT can substantially improve survival rates in patients with severe alcohol-related hepatitis. For example, in one small pilot study, single-donor FMT increased the 28-day survival rate to 100% (vs. 60%) and the 90-day survival rate to 54% (vs. 25%) (Sharma et al., 2022). More recently, a large-scale RCT confirmed that the 90-day survival rate was 75% with daily FMT (given for 7 days) compared to 56% with conventional steroid treatment (Pande et al., 2023); the benefit of FMT was also highly significant (hazard ratio 0.53, p = 0.044), and the infection-related mortality rate among FMT-treated patients was much lower (Pande et al., 2023). Systematic reviews also confirm that FMT can significantly improve short-term survival in severe alcoholic hepatitis. A meta-analysis calculated pooled odds ratios of ∼2.9 at 28 days and ∼3.1 at 90 days for survival with FMT vs. standard care (Taha et al., 2024). Importantly, long-term mortality between FMT-treated and control patients appears to converge later on, but an early survival benefit is evident. The presumed mechanism behind FMT’s efficacy is restoration of the gut barrier and reduction of endotoxemia, which in turn mitigates the inflammatory drive on the liver. Overall, FMT has emerged as a promising new therapy that can substantially improve short-term outcomes in patients with severe alcoholic hepatitis when added to conventional treatment regimens (Pande et al., 2023; Taha et al., 2024).

Despite these encouraging short-term results, FMT is not a cure for advanced cirrhosis. The current literature has not reported any evidence of fibrosis regression or long-term recovery of liver function following FMT administration. Meta-analyses show that the potential survival benefit from FMT appears limited to the initial months after treatment, with no benefit observed at 6–12 months (Taha et al., 2024). Even pooled analyses show that by 6 months after treatment, the survival curves of those given FMT and controls are once again statistically identical (Taha et al., 2024). Such results suggest that, while FMT may allow for time to be gained through the resolution of gut-source inflammation, established liver scarring is not treated by the approach. It is also worth noting that much of the variability between studies may relate to trial design parameters (e.g., donor characteristics, methods of delivering FMT to the patient) (Taha et al., 2024). Clearly, larger controlled trials are needed to determine whether any long-term improvements can be achieved with repeat FMT dosing or adjunctive therapies.

Compared with probiotics, FMT induces a much broader-scale reorganization of the gut microbiome. FMT (non-standard probiotic supplementation) has been shown to confer substantially greater short-term survival benefits in patients with severe AH (Pande et al., 2023). Probiotics have been shown to reduce ALT and AST levels by an average of about 10–20 U/L (Xiong et al., 2024). In contrast, FMT introduces about 20–30 previously undetected beneficial taxa. In one demonstration of this, 23 engrafted commensal species were present by the 28-day mark post-treatment (including multiple undetermined species belonging to the Lachnospiraceae and *Prevotella* genera). At the same time, a subsequent decline in enterobacteriaceae was also measured (Pande et al., 2023). The extreme level of diversity “reset” to near baseline levels following FMT, which may explain its markedly greater influence on endotoxin levels and downstream inflammation. However, important limitations to its use include the need for rigorous donor evaluation and the requirement for more invasive methods of treatment administration. Nevertheless, two meta-analyses of FMT for acute ALD have recently reported its efficacy and emphasized the need to develop standardized protocols and conduct confirmatory trials (Pande et al., 2023; Taha et al., 2024). By contrast, probiotic therapies are simpler and generally well-tolerated (Amadieu et al., 2022; Rattan and Shah, 2022). However, no consistent evidence of the efficacy of probiotic treatment for severe ALD has yet been published (Xiong et al., 2024). Future studies that compare microbiome-based treatments head-to-head (e.g., multi-species synbiotics vs. FMT) can ultimately be designed to identify which treatment approach is most effective and safest for patients with ALD.

A major advantage of FMT is that it delivers an entire microbial community in one treatment, effectively knocking down multiple pathobionts simultaneously. In a very severe alcoholic hepatitis study, for example, FMT recipients gained about 23 new commensal species while rapidly (within 1 month) reducing the levels of certain pathogenic genera (e.g., *Campylobacter*, *Weissella*) (Pande et al., 2023). The breadth of engraftment seen in this experiment far surpasses anything a single-strain probiotic could hope to accomplish. Yet FMT also poses some challenges. Stool preparations can vary widely from donor to donor, and without established universal manufacturing standards, FMT products can be highly heterogeneous. Donor screening practices are still evolving, and ensuring that each FMT product is safe to use (i.e., free of potential infection transmission) presents challenges. The delivery process itself is invasive (via endoscopy, nasoduodenal tube, or colonoscopy), so it may not be accessible to all patients seeking treatment. Thus, for the field to advance further, it is necessary to develop standardized methods to stabilize the donor selection and processing process to enhance consistency from one product to the next. Yet by effectively “resetting” the microbiome, FMT directly addresses gut-derived endotoxemia, which sets it apart from the more limited approach of using probiotics.

The novelty and invasive nature of FMT also raise important ethical and regulatory concerns. Patients must be fully informed of the potential risks, including the rare transmission of infection and the possible psychological implications of the procedure. Indeed, surveys suggest that some patients find the idea of FMT to be distasteful, underscoring the need for thorough counseling and perhaps the development of less invasive approaches (Skryabin, 2025). Centers must implement a consistent approach to donor screening to limit risk where possible, and must have a policy to ensure consistency (to prevent this from becoming a procedure restricted to a limited number of centers due to cost). As microbiome-based therapies diversify (including engineered microbes and personalized phage cocktails), regulation will also need to evolve. There must be a framework for evaluating novel microbiome-based therapies to ensure patients remain safe and that their rights to privacy and self-determination are respected. In short, FMT and related developments must be aligned with an evolving ethical framework that protects the patient (Skryabin, 2025).

In summary, there is growing evidence that FMT is a useful adjunctive treatment for severe alcoholic hepatitis, although larger studies are needed to confirm this. Recent meta-analyses suggest a short-term survival benefit from FMT, while calling for multicenter RCTs with adequate sample sizes and standardized protocols to confirm these findings (Pande et al., 2023; Taha et al., 2024). Because FMT resets the microbiome broadly without targeting specific pathogens, researchers are pursuing more precise approaches. For instance, bacteriophage (virus) cocktails aimed at specific ALD-related bacteria – such as cytolytic *E. faecalis* or pathogenic *E. coli* strains – have shown promise in preclinical ALD models (Duan et al., 2019). In the next few years, we can expect to see these targeted microbiome-based treatments (including engineered probiotics) tested in clinical trials for patients with ALD. The combination of these treatments with nutritional supplementation and abstinence counseling will be the most comprehensive treatment strategy for addressing ALD.

### 5.3 Customized bacteriophage therapy

Bacteriophage (phage) therapy is emerging as a precision tool for eliminating a subset of gut pathogens known to contribute to the development of ALD. In a groundbreaking, preclinical study, Duan et al. (2019) used oral phage cocktails to delete a cytolytic strain of *E. faecalis* in humanized ALD mice. They prevented the mice from suffering liver damage from ethanol (Duan et al., 2019). The levels of their liver enzymes (ALT/AST) were even normalized, and the mice’s liver histology was preserved. Thus, knocking out a single causative pathogen appears to be an effective means of halting the progression of liver damage in ALD in a model organism. Other studies have identified bacteria associated with hepatocyte injury and the progression of ALD (Philips et al., 2022). For example, many ALD patients who have developed this disease have been found to harbor a specific strain of *E. coli*, and this strain can evade the immune system only with the help of a capsular gene called *kpsM.* Researchers have used a small molecule that blocks its action to improve models of liver damage (Yang et al., 2025). Thus, to eliminate a subset of microbes that contribute to ALD development, phage therapy could be a promising treatment. However, to date, this strategy remains largely theoretical in humans.

Phage therapy is attractive because a phage (or a defined cocktail of phages) can selectively lyse a single bacterial species while preserving the rest of the gut microbiota (Suh, 2021; Strathdee et al., 2023). This approach does not cause the widespread disruption of the microbiome that broad-spectrum antibiotics can, and it bypasses traditional antibiotic resistance mechanisms. Furthermore, it may be possible to select or engineer phages that target multiple strains of bacteria implicated in ALD and that can penetrate the biofilms that protect these bacteria. Numerous practical obstacles remain, however. Challenges include bacterial phage-resistance (necessitating phage cocktails or sequential treatments), delivery of phages to the gut (oral doses may be inactivated by gastric acid without special encapsulation; rectal delivery is invasive), and the scale-up of high-quality phage production under regulatory standards (Ganeshan and Hosseinidoust, 2019)**.** At present, clinical evidence for phage therapy in ALD remains very limited, with most data coming from mechanistic and animal studies (Fujiki and Schnabl, 2023; Strathdee et al., 2023). Until these obstacles are overcome, phages can only be viewed as a promising research tool rather than a treatment (Suh, 2021).

High-throughput sequencing has revealed specific bacteria associated with ALD, providing a rationale for precision phage therapy targeting these pathogens while sparing the rest of the microbiome (Cabré et al., 2023). Indeed, phage treatments in alcohol-fed animal models have reduced liver damage, reinforcing the potential clinical relevance of this approach (Suh, 2021). Synthetic biology advances even allow the engineering of ‘designer’ phages with enhanced capabilities. However, such constructs have not been tested in ALD and raise additional concerns (e.g., ensuring efficacy, avoiding immune neutralization, and preventing horizontal gene transfer) (Cabré et al., 2023). In summary, current evidence on phage therapy in ALD is confined to mechanistic and animal studies (Fujiki and Schnabl, 2023). Rigorous translational research and clinical trials will be required, along with appropriate regulatory frameworks, before this strategy can be translated into routine ALD treatment (Strathdee et al., 2023).

### 5.4 Postbiotics, diet, and adsorbents

Postbiotics (non-viable microbial preparations containing bioactive components and metabolites) are emerging as a practical and promising candidate for the treatment of ALD due to their stability and safety (they contain no living organisms, thus cannot colonize or mutate in the host) (Dukić et al., 2023). Preclinical studies show that certain *Lactobacillus*-derived postbiotic formulations can restore gut barrier integrity and reduce alcohol-induced liver inflammation, possibly by modulating bile acid–FXR signaling (Liu C. et al., 2024). Initial clinical data are encouraging: in a recent Phase II trial, a *Lactobacillus acidophilus* lysate was well-tolerated and showed a non-significant trend toward improved fibrosis markers in advanced ALD (Hansen et al., 2025). These preliminary results warrant well-powered, large-scale trials to determine postbiotics’ true efficacy in ALD, optimal formulations, and long-term outcomes.

Lifestyle factors (diet and environment) clearly influence the gut microbiome and its interaction with the liver (Jung et al., 2022). For instance, in animal models fermentable fiber intake protected against alcohol-induced liver injury by restoring microbial balance (higher SCFA levels, improved intestinal barrier) and reducing inflammation. On the other hand, the specific effects of manipulating single bacterial species and the role of bile acid–FXR pathways have so far been demonstrated only in animal studies (Jung et al., 2022; Sosnowski and Przybylkowski, 2024), not yet in humans. Moreover, diets rich in plant-derived polyphenols have been associated with greater microbiome diversity and lower endotoxemia, whereas diets high in refined sugars and fats have the opposite effect–they disrupt the gut barrier and exacerbate liver inflammation (Dukić et al., 2023; Hsu and Schnabl, 2023). Beyond dietary modulation, various microbiome-targeted approaches are being investigated. For example, postbiotic formulations derived from probiotic strains have shown beneficial effects on gut barrier integrity and alcohol-induced hepatic inflammation in animal models. In contrast, early human data are limited and mainly demonstrate safety and tolerability (Dukić et al., 2023). Device-based interventions that sequester gut-derived toxins are also being explored as a novel strategy for treating ALD. Oral activated carbon formulations, such as CARBALIVE beads, are currently being tested in preclinical studies for their efficacy in advanced liver disease. However, early-phase human studies have reported only on safety and tolerability, with no findings on clinical efficacy (Macnaughtan et al., 2021). All in all, dietary, microbiome-focused, and device-based interventions represent a spectrum of strategies to modulate the gut in ALD. Backed by robust mechanistic and preclinical findings, these methods nonetheless demand carefully crafted clinical trials to verify their effectiveness, support their incorporation into routine care, and evaluate their sustained outcomes.

In conclusion, comprehensive supportive therapies remain an essential component of ALD management, alongside microbiome-targeted therapies. Alcohol abstinence and adequate nutrition are key components of the treatment for ALD and are consistently associated with benefit (Ayares et al., 2022). Dietary approaches that emphasize anti-inflammatory and well-balanced food patterns may favorably affect the gut-liver axis, though at this point the supporting evidence is largely experimental or observational (Hsu and Schnabl, 2023). Physical exercise is beneficial for metabolic health and can modify gut microbiota composition in liver metabolic diseases (Cullen et al., 2023). However, evidence for the view that exercise-induced changes in the gut microbiota contribute to the effects observed with the reversion of ALD symptoms has not yet been sufficiently investigated. Other emerging approaches for targeting the microbiome in liver diseases include next-generation probiotics and engineered commensal organisms. *Faecalibacterium prausnitzii* is a major butyrate-producing commensal and has anti-inflammatory effects in preclinical models. However, direct evidence for its involvement in the treatment of ALD has yet to be published (Hsu and Schnabl, 2023). Taking all available information into account, the literature supports a multifaceted treatment approach that encompasses lifestyle modifications, microbiome-targeted therapies, and pharmacological treatments, with the caveat that further clinical studies will be necessary to define the additive or synergistic effects that may be achievable.

### 5.5 Supportive therapies

Management of ALD has generally focused upon supportive therapies that aim to address secondary complications rather than directly impact the disease process. Yet high-quality evidence from randomized controlled trials supports the use of non-absorbable antibiotics (such as rifaximin) for the treatment of hepatic encephalopathy, since these agents reduce intestinal ammonia levels and can lead to significant improvements in neurocognitive function. However, the application of these agents (and their effects) is restricted to the management of secondary complications rather than affecting liver disease itself (Zacharias et al., 2023). In contrast, moderate-quality evidence supports the use of antioxidant therapy in selected cases. For instance, N-acetylcysteine has been demonstrated to exert short-term benefit in the treatment of severe alcoholic hepatitis, whereas evidence for vitamin E, silymarin, and ursodeoxycholic acid is inconsistent or negative (Nguyen-Khac et al., 2011; Gillessen and Schmidt, 2020). Conversely, a randomized trial of TNF-α blockade failed to show benefit and instead increased the risk of adverse outcomes in patients with alcoholic hepatitis (Boetticher et al., 2008). Emerging metabolic or molecularly targeted therapies for ALD are still in preclinical or experimental stages. For now, the cornerstones of management remain sustained alcohol abstinence, nutritional support, and comprehensive supportive care–interventions whose importance is well-supported by longitudinal clinical studies (Ayares et al., 2022; Anouti et al., 2024). Gut-focused treatments will likely complement (not replace) these standard measures. Even combined regimens that integrate microbiome therapy with conventional care are considered experimental until tested in properly powered trials (Rattan and Shah, 2022).

Gut–liver–directed therapies in ALD may also intersect with the gut–brain axis. Alcohol-induced dysbiosis in the gut has been associated with systemic inflammation, neuroinflammation, and increased alcohol craving in observational and mechanistic studies (Du et al., 2022; Grodin et al., 2024). Consistent with this potential intersection, two small early-phase clinical studies found that FMT reduced self-reported alcohol craving and consumption within days to weeks of treatment initiation. FMT-treated subjects also showed improvement in cognitive function and quality of life, and reductions in LPS-related markers and IL-6 (Bajaj et al., 2020; Bajaj et al., 2021). While preliminary, these findings suggest that reducing gut-derived inflammation may confer benefits for the brain. Other microbiome-modulating treatments (probiotics, prebiotics) and dietary changes have been postulated to affect neurotransmitter levels (e.g., GABA, dopamine); however, evidence for these effects is mostly preclinical or speculative, with no strong clinical evidence to date (Bernhardt et al., 2024). Thus, treatment of ALD should continue to include dedicated addiction counseling and psychosocial support in addition to any medical interventions, as recommended by clinical guidelines (Arab et al., 2022). Potential future clinical studies of gut-directed therapies for ALD could incorporate validated measures of alcohol craving, mental health, and neurocognitive function to understand their global effects.

## 6 Discussion

This review supports the view that microbiota dysbiosis plays a core role in ALD pathogenesis (Zhang D. Y. et al., 2023) and highlights recent mechanistic and therapeutic insights as well as developments in microbiome-targeted interventions. However, many aspects of the gut-liver axis in ALD remain in the early stages of research, and further studies are required to clarify the disease process and translate this knowledge into practice. The most notable alterations in the ALD gut microbiome are bacterial dysbiosis, fungal overgrowth, and virome expansion. These have been associated with functional disruptions of the gut-liver axis, including the loss of beneficial commensal bacteria, increased translocation of microbial toxins, and enhanced inflammatory responses (Table 1). Taken together, the current evidence suggests that alterations in the gut microbiome are active drivers (not passive bystanders) of ALD progression (Zhang D. Y. et al., 2023; Grodin et al., 2024), which further supports the concept of targeting the gut in the treatment of this disease.

**TABLE 1:** Overview of alterations in gut microbiota in alcohol-associated liver disease (ALD).

Microbial group	Category/Item	Specific Microbes/Components	Change and functional impact in ALD	Associated mechanisms	Therapeutic implications	Key references
Bacteria	Beneficial commensals	*Bifidobacterium* spp.; *Faecalibacterium prausnitzii* (*F. prausnitzii*)*; A. muciniphila*	Reduced *Bifidobacterium* spp. And *F. prausnitzii*→ decreased colonization resistance, lower SCFA availability, and impaired barrier function. Reduced *A. muciniphila* → thinner mucus layer, increased permeability (“leaky gut”), and greater microbial/endotoxin translocation to the liver	Loss of barrier-supporting metabolites (e.g., SCFAs) and mucus maintenance increases intestinal permeability and promotes inflammation	Microbiota restoration strategies (e.g., probiotics/synbiotics, fecal microbiota transplantation) may help replenish beneficial taxa. Supplementation with *A. muciniphila* improved the mucus barrier and reduced alcohol-induced liver injury in mouse models	Grander et al. (2018); Li et al. (2025)
Bacteria	Pathobiont expansion	Enterobacteriaceae *(*e.g., *E. coli); E. faecalis* (cytolysin-positive strains)	Overgrowth of gram-negative enterobacteriaceae and cytolysin + *E. faecalis* increases endotoxin/toxin exposure and is linked to hepatic inflammation and severe alcoholic hepatitis	Barrier disruption enables LPS translocation and TLR4 pathway activation; cytolysin directly injures hepatocytes	Investigational approaches include bacteriophages targeting cytolysin + *E. faecalis*, microbiota replacement (FMT) in severe alcoholic hepatitis, and strategies to reduce LPS-producing bacteria or dampen LPS signaling	Duan et al. (2019); Yang et al. (2025)
Bacteria	Ethanol-producing bacteria	*Klebsiella pneumoniae*; certain *E. coli* strains	Endogenous microbial ethanol production between drinking episodes may sustain intestinal ethanol levels and exacerbate hepatic injury and inflammation	Persistent low-level ethanol exposure contributes to hepatocyte toxicity and pro-inflammatory signaling	Potential future strategies: Reduce ethanol-producing strains (precision probiotics/synbiotics or selective antimicrobials) to lower endogenous ethanol burden	Andaloro et al. (2025); Park et al. (2021)
Fungi	Reduced fungal diversity	Commensal gut fungi (mycobiome)	Chronic alcohol exposure is associated with decreased fungal diversity, which may weaken mucosal defenses and facilitate opportunistic overgrowth; combined bacterial–fungal dysbiosis can amplify permeability and inflammation	Reduced mycobiome diversity may impair barrier defense and immune homeostasis, contributing to gut permeability and inflammatory activation	Broad microbiome restoration (e.g., FMT) may partially correct multi-kingdom dysbiosis; no established therapy specifically restores fungal diversity	Lang et al. (2020b)
Fungi	*Candida* overgrowth	*C. albicans*; fungal components (β-glucan, candidalysin)	Alcohol-related barrier injury permits *C. albicans *bloom/invasion. Candidalysin and fungal translocation (e.g., β-glucan) amplify hepatic inflammation. Elevated antifungal markers (e.g., ASCA, β-glucan) correlate with worse outcomes in ALD.	β-glucan translocation activates Dectin-1 signaling and IL-1β release, promoting Th17 responses (IL-17) and neutrophil-mediated tissue injury; candidalysin can directly damage hepatocytes.	Non-absorbable antifungals reduced intestinal *Candida *load and β-glucan levels in models, attenuating IL-1β/Th17 inflammation. FMT has also been explored in steroid-refractory severe alcoholic hepatitis, likely by correcting bacterial and fungal dysbiosis	Chu et al. (2020); Zeng et al. (2023)
Viruses	Gut viruses (virome) expansion	Expanded bacteriophages and eukaryotic viruses (e.g., Parvoviridae, Herpesviridae)	ALD—especially severe alcoholic hepatitis—has been associated with increased gut virome diversity. Shifts in bacteriophages and eukaryotic viruses may reshape bacterial communities, promote endotoxin release, and worsen barrier dysfunction; Herpesviridae abundance has been linked to disease severity and mortality	Alcohol-associated immune dysregulation may trigger viral reactivation; phage-mediated bacterial lysis can alter microbiota composition and increase endotoxin exposure	Emerging concepts include fecal virome transplantation (FVT) and targeted phage therapy (e.g., against cytolysin + *E. faecalis* in preclinical models)	Jiang et al. (2020); Hsu et al. (2022)

.

### 6.1 Deepening mechanistic insights and causal validation

Recent mechanistic studies have defined multiple mechanisms by which gut dysbiosis causes liver injury in ALD. Microbial metabolites and virulence factors from dysbiotic communities have direct toxic effects; for example, translocation of bacterial endotoxin and fungal β-glucan into the portal circulation activates hepatic TLR4 and Dectin-1, which, in turn, initiate inflammatory signaling (Chu et al., 2020). Similarly, microbial toxins such as *E. faecalis* cytolysin and *C. albicans* candidalysin have been shown to kill hepatocytes directly (Duan et al., 2019; Chu et al., 2020). In parallel, the depletion of beneficial microbes in ALD compromises the integrity of the intestinal barrier, enabling additional microbe-derived products to escape into the liver. These findings demonstrate that gut microbiota abnormality is a causal mechanism of ALD development rather than simply an association (Wang et al., 2021).

Even with these advances, however, proving direct causality for the dysbiotic state remains a challenge (Wang et al., 2021). The correlations underlying the association between the microbiome and ALD have not yet been sufficiently validated to support claims of causality (Wang et al., 2021). Hence, upcoming investigations should prioritize thorough confirmation of how specific microbial populations or their metabolites may causally contribute to ALD (Wang et al., 2021). For example, germ-free animal models colonized with defined (or engineered) microbial communities and/or metabolomes can be used to assess the individual contribution of specific microbes or their byproducts to alcohol-related liver damage (Hendrikx et al., 2019). This type of experiment can test the contribution of specific microbes (or their metabolites) to the development of alcohol-induced liver injury (Hendrikx et al., 2019). In the case of humans, humanized microbiome mouse models plus co-culture systems incorporating organoids may hold the key. Such models enable testing how the human gut microbiome influences liver immune function in a controlled setting (Bajaj and Hylemon, 2018). Finally, another promising avenue for research exists in the yet-untapped field of immunometabolism in ALD. It remains to be determined how changes in the microbiome induced by alcohol affect the metabolic reprogramming of immune cells that accumulate in the liver and contribute to liver inflammation (Jung et al., 2022; Chen M. et al., 2023; Dukić et al., 2023). Insight along these lines can not only deepen our understanding of how microbiome changes contribute to the pathogenesis of ALD, but it can also identify additional targets for therapy.

### 6.2 Technological and methodological innovations

Emerging technologies are opening new pathways to study the ALD-microbiome connection. Single-cell “omics” are being applied to the host and gut microbiota, allowing the detailed resolution of the dynamics of the intestinal inflammatory microenvironment (Liu et al., 2024b). Metagenomic sequencing with next-generation sequencing technologies, culturomics, and metatranscriptomics enable deeper analysis of the functions and regulation of microbes within complex gut communities (Magdy Wasfy et al., 2025). Metabolomics directly quantifies the metabolites produced by the microbiota, though its interpretations are confounded by diet and environmental effects; complementary methods, such as metabolic flux analysis, can pinpoint specific microbial contributions to metabolic flux (Kuo et al., 2025)**.** Systems biology models that integrate multi-omics data sets (genomic, transcriptomic, metabolomic, …) are also being developed (though they are all at very early stages). Machine learning algorithms can extract meaningful information from high-dimensional data sets on networks linking microbes and their metabolites that drive ALD progression (Gao et al., 2021). For example, computational models have already revealed clusters of microbial genes and metabolites associated with fibrosis or with treatment response, which can be tested for inclusion as biomarkers (Ghosh et al., 2025b).

Standardization and reproducibility are emerging themes as the field becomes more saturated. Standardized 16S rRNA sequencing data analysis pipelines across studies and control of potential confounders (diet, medications, alcohol) will all enhance the ability to make valid comparisons (Thiele et al., 2024). All this will ensure that what is seen is associated with the disease in question rather than with something else. Better bioinformatics tools, combined with growing collaborations between microbiologists, hepatologists, and data scientists, will also help to extract relevant insights from this complex multi-omics data. In particular, the use of artificial intelligence (AI) and machine learning (ML) techniques has already shown impressive returns (Gao et al., 2022; Thiele et al., 2024). AI algorithms can sort through millions of noisy data points looking for patterns and associations that may remain hidden to human investigators (Zheng et al., 2025). For example, the new study on ALD and related hepatic disorders uses AI-based multi-omics analysis to identify microbiome-associated biomarkers and disease subtypes, suggesting that this computational modeling approach could reveal new subtypes and treatment targets (Ghosh et al., 2025b). AI-fueled ML algorithms that integrate metagenomic, metabolomic, and clinical data have already increased predictions for ALD progression risk for individual patients (Gao et al., 2022). Importantly, tools like these are opening the door to personalized interventions. Predictive models could soon enable treatment recommendations that account for an individual’s dysbiosis profile, such as a patient-specific phage therapy or probiotic formula (Yang et al., 2026). To wrap things up, harnessing advanced tools—from single-cell profiling to multi-omics fusion and AI-based simulations—has been speeding breakthroughs in ALD studies, while addressing stubborn obstacles to nailing down causal links and pinpointing viable treatments.

### 6.3 Challenges in clinical translation

To translate findings from the microbiome field into effective ALD therapies, several hurdles need to be overcome. The existing clinical literature is inconsistent and heterogeneous, in part because earlier studies were often too small and varied in methodology, yielding inconsistent results (Grodin et al., 2024). Well-powered, multicenter studies with rigorous, standardized protocols are required to yield reliable, generalizable findings. The clinical utility of any intervention (e.g., probiotics, FMT) for the treatment of ALD remains unproven, as no completed large-scale randomized controlled trial (RCT) has been conducted to evaluate it (Wolstenholme et al., 2024). Short-term treatment with probiotics or prebiotics produces only marginal improvements in measures of gut barrier function and liver enzyme activity, and its long-term clinical relevance has yet to be established (Xiong et al., 2024). Similarly, though FMT shows conceptual promise and links to enhanced short-term outcomes in severe alcoholic hepatitis, it remains an invasive intervention, and its lasting impact on liver recovery is not fully clear yet (Taha et al., 2024). Bacteriophage therapy is an emerging new treatment approach. Preliminary results from animal studies (for example, a phage that targets cytolytic *E. faecalis* prevents ALD in mice) (Mendes et al., 2022) suggest its promise. However, it is also still in the experimental phase with no data yet available for clinical use. Postbiotics - non-viable microbial preparations that contain bioactive metabolites-are an alternative, theoretically safer and more stable than live biotherapeutics. Initial research shows that postbiotic formulations (e.g., *Lactobacillus fermentum*) can restore barrier function and counteract alcohol-induced inflammation in ALD models (Liu C. et al., 2024). However, a recently completed phase 2 trial of a *Lactobacillus*-derived postbiotic preparation for the treatment of advanced ALD showed good tolerability and a trend toward improvement in fibrosis markers. Still, this finding was not statistically significant (Hansen et al., 2025). These findings show promise, but clinical evidence for postbiotics remains limited. Larger, well-designed studies are required to validate their effectiveness and to define the best formulation and dosage regimen.

High-quality RCTs that include diverse ethnic and geographic populations are especially needed to demonstrate that the approach is applicable across populations (Grodin et al., 2024). However, individual patient characteristics (genetic background, diet, baseline microbiome composition) also play a large role in the response to therapy. The microbiota of each patient is unique, so one size does not fit all when it comes to probiotics or phages. The most effective treatment approach will likely be a personalized treatment using probiotic strains or phage cocktails tailored to the individual case based on their unique dysbiosis profile (Zhang D. Y. et al., 2023). Development of rapid-turnaround clinical microbiome diagnostic tests (e.g., sequencing-based tests) and AI-empowered treatment guidance could help make individualized treatment approaches more accessible (Ghosh et al., 2025b). For instance, an AI model could predict which patients would respond to a given probiotic treatment or identify the correct bacteriophages to target a patient’s overpopulated pathobionts.

Yet another translational challenge is the issue of novel treatment safety and standardization. FMT’s transition from an experimental to an approved treatment will require addressing donor screening, stool processing protocols, and regulatory frameworks to prevent adverse reactions (Ma L. et al., 2025). The same applies to phage therapy if it is to become a clinical treatment; there will need to be quality control and regulatory considerations to address issues such as phage purity, resistance, and patient immune reactions (Strathdee et al., 2023). Beyond these microbiome-targeted therapies, however, lifestyle modifications are another corner of the ALD management landscape. Thus far, alcohol avoidance, improved nutrition, and exercise have all been shown to beneficially affect the gut-liver axis (evidence for the benefit of exercise in ALD is only beginning to emerge) (Anouti et al., 2024). However, the question of whether the greatest benefit accrues from integrating these two intervention areas remains to be studied. For example, if “early treatment” protocols incorporating optimization of diet and possibly the use of prophylactic probiotics for those at greatest risk (for example, heavy drinkers with metabolic risk factors) can prevent or delay the onset of disease even in those who have already begun to accumulate several risk factors (Jung et al., 2022). Beyond these preclinical studies, however, the question of how to translate promising data from the lab to human patients is, first and foremost, a safety challenge. Much of what is known about the role of the microbiome and its associated microbes in ALD has been learned from studies using animal models. Such models are, by definition, imperfect and do not account for the complexity of the human gut microbiome or the functioning immune system (Hyun et al., 2022). This implies the need for target validation in human-relevant models: organoid systems, humanized-microbiota mice, and well-designed clinical trials to confirm that targets identified in mice make clinical sense in humans (Hyun et al., 2022; Feng et al., 2024). This next step will again require combined efforts and expertise from researchers across multiple fields. Hepatologists, microbiologists, and immunologists all need to be at the same table as translational studies are designed. Shared data will all but guarantee rapid development of safe, effective microbiome-targeted therapies for ALD.

### 6.4 Socioeconomic factors and global disparities

Socioeconomic factors, as it happens, influence both the incidence and progression of ALD through their indirect effects on the gut microbiome. Lower socioeconomic status (SES) is associated with higher levels of alcohol consumption, lower quality of diet (e.g., less fiber and more processed foods), and less access to healthcare, all factors that predispose individuals to gut dysbiosis and ALD (Bishehsari et al., 2023; Zhu et al., 2023). Moreover, economic hardship and chronic psychosocial stress (more common in lower-SES environments) can disrupt the gut-brain axis via neurohormonal pathways, potentially further disrupting the microbiota (Zhu et al., 2023). These findings suggest that social determinants of health may shape the gut microbial community, predisposing individuals to ALD. There are stark global inequalities in the availability of microbiome-targeted treatments for ALD, evident in the unequal distribution of FMT research and treatment activity around the world, which remains heavily concentrated in high-income settings (Olesen et al., 2020). However, relevant public health interventions may reduce the burden of liver disease caused by alcohol abuse. For example, campaigns to promote the consumption of foods with beneficial effects on the gut microbiome (e.g., those that are high in fiber and plant-based) and to limit excessive drinking at a population level may be effective in maintaining a healthy level of microbiome and liver function, reducing the risk of developing ALD (Li et al., 2021; Weichselbaum et al., 2025). Achieving fair access to microbiome therapies will require scientific advances in tandem with targeted policies, education, and community efforts – all aimed at overcoming persistent socioeconomic barriers**.**

Future research needs to address several key scientific questions to proceed.

1. Global Microbiome Differences: What are the population-specific gut microbiome features that influence ALD risk or progression in Eastern vs. Western populations (Koponen et al., 2025)? Are there ethnic or geographic differences in the gut microbiome that could inform population-specific prevention strategies?
2. Microbiome Evolution During Disease: How does the gut microbiome composition evolve over the course of ALD, and can changes serve as early predictors of disease progression (Grodin et al., 2024; Koponen et al., 2025)? Longitudinal studies are required to determine whether shifts in the microbiome precede or follow disease-related events.
3. Optimal Intervention Timing: What is the most appropriate stage of disease to implement microbiome-based interventions, and how should different therapies be combined for optimal benefit? For example, when should probiotics be given for prevention, *versus* FMT for acute alcoholic hepatitis (Jung et al., 2022; Ma J. et al., 2025)? Establishing consensus on the right intervention at the right time will ensure that patients receive the maximum benefit from these interventions.
4. Combination Treatment Strategies: Can different types of interventions (probiotics, prebiotics, FMT, phages, postbiotics, *etc.*) be safely combined to achieve additive effects (Bajaj et al., 2022)? Establishing a combination treatment that is more effective than any one treatment alone will be necessary for the development of microbiome-based therapies for ALD.
5. Multi-omics for Causal Insights: How can multi-omics approaches be utilized to identify causal relationships between the gut microbiome and ALD progression, and to develop predictive models for clinical decision support (Gao et al., 2022)? Advanced analytics combined with genomics, metabolomics, and metagenomics may uncover robust biomarkers and therapeutic targets while also enabling meaningful patient stratification.
6. Standardization of Approaches: How can treatment protocols for microbiome-based interventions in ALD (e.g., FMT donor criteria, probiotic strain selection and dosing) be standardized to improve reproducibility and facilitate clinical adoption (Ma L. et al., 2025)? For example, would implementing unified screening criteria for FMT donors or using a standardized probiotic cocktail across trials increase comparability between studies and allow identification of true efficacy signals?

Addressing these questions will guide future research aimed at harnessing the microbiome for personalized ALD treatment. ALD research has made considerable progress in understanding the gut-liver axis and developing microbiome-based therapies; yet ongoing research and interdisciplinary collaborations are required to overcome current gaps. By closing these knowledge gaps and surmounting translational barriers, the field can move toward the clinical realization of microbiome-oriented interventions to improve prevention and treatment of ALD.

## 7 Conclusion

The gut microbiome plays a central role in the occurrence and progression of ALD. Research indicates that ALD develops through a combination of alcohol-induced gut microbiome disruption, intestinal barrier breakdown, and immune dysregulation. The gut microbiota and their metabolic byproducts function as disease markers, enabling researchers to develop new therapeutic approaches. There have been promising outcomes with some microbiome-based treatments (probiotics and FMT) in ALD patients, and even experimental approaches like bacteriophage therapy show potential in preclinical studies. These therapeutic methods require additional research and testing before they can be widely implemented in clinical practice. Notably, despite extensive profiling, a definitive diagnostic microbiome signature for ALD is still lacking, reinforcing the need for composite biomarkers and personalized approaches (as discussed). Ongoing research needs to combine multi-omics data analysis with mechanism-focused studies to link current understanding with practical treatment development. Expanding knowledge of the gut-liver axis is expected to lead to the clinical adoption of microbiome-based, individualized prevention and treatment approaches for ALD patients. The gut microbiome offers a novel therapeutic avenue for ALD, complementing standard medical therapies to address gut-liver axis dysfunction. Probiotics may help reduce inflammation while strengthening the gut barrier. FMT has demonstrated the potential to improve short-term survival in patients with advanced alcoholic hepatitis. The development of microbiome-based treatments is promising but will require standardization and validation through large-scale randomized controlled trials to reach its full potential. Achieving this potential will depend on evidence-based research and teamwork across medical disciplines. A strong interdisciplinary partnership between hepatologists, microbiologists, immunologists, geneticists, nutritionists, and ethicists is essential to create safe and effective personalized microbiome-targeted treatments for ALD. In conclusion, ALD arises from the combined effects of host-intrinsic and environmental factors, and maintaining gut microbial balance will be an important component of ALD prevention and therapy.
